# Solitary Fibrous Tumor With a 12-Year Recurrence Interval in a Female Patient: A Case Report

**DOI:** 10.7759/cureus.101592

**Published:** 2026-01-15

**Authors:** Diana Laura Rodríguez Carrillo, Begoña Llaca Morfin, Moises Brener Chaoul, Carlos D Robles Vidal

**Affiliations:** 1 General Surgery, Hospital Angeles Lomas, Huixquilucan, MEX; 2 Surgical Oncology, Hospital Angeles Lomas, Huixquilucan, MEX

**Keywords:** follow-up, mediastinum, metachronous tumor, recurrence, solitary fibrous tumor

## Abstract

Solitary fibrous tumor (SFT) is a rare mesenchymal neoplasm characterized by unpredictable biological behavior, with the potential for late recurrence even in tumors initially classified as low risk. We report the case of an 83-year-old woman with a history of complete resection of a right thoracic SFT 12 years earlier, who was found on surveillance imaging to have a new anterior left mediastinal mass. Positron emission tomography-computed tomography (PET-CT) demonstrated low metabolic activity (maximum standardized uptake value (SUVmax) 2.32). Surgical exploration revealed a 6.5 × 5 cm lesion adherent to the pericardial pleura, which was completely resected. Histopathologic examination showed a spindle cell neoplasm with a patternless architecture, without hypercellularity, necrosis, or pleomorphism. Immunohistochemistry demonstrated strong nuclear positivity for signal transducer and activator of transcription 6 (STAT6) and diffuse CD34 expression, with negative S100 protein and muscle markers. Risk stratification using the Demicco model categorized the tumor as low risk. Review of the original tumor resected in 2013, which measured 19.3 × 16.2 cm, revealed similar histologic features and an intermediate-risk score. The long disease-free interval raises the diagnostic dilemma of late recurrence versus a metachronous SFT, a distinction that remains challenging due to shared morphologic and molecular characteristics. This case underscores the limitations of metabolic imaging in indolent SFTs and highlights the importance of complete surgical resection and long-term, potentially lifelong, surveillance.

## Introduction

Solitary fibrous tumors (SFTs) are exceedingly rare mesenchymal neoplasms composed of spindle cells arranged in a patternless architecture interspersed with a thin, branching (*staghorn*) vascular network [1]. They represent less than 2% of all soft tissue tumors and are estimated to have an annual incidence of approximately 0.2 cases per 100,000 individuals [2]. The diagnosis of SFT currently relies on the identification of NAB2-STAT6 gene fusions, with nuclear signal transducer and activator of transcription 6 (STAT6) immunohistochemistry serving as a reliable surrogate marker. SFTs may arise at virtually any anatomic site, either deep or superficial [3].

Clinically, SFTs typically present as slow-growing masses that may reach considerable size before producing symptoms; in more than half of cases, tumors exceed 10 cm at the time of diagnosis [1]. Although the majority of SFTs exhibit indolent behavior, approximately 10%-30% demonstrate malignant potential, with local invasion, recurrence, and distant metastasis [4]. The risk of recurrence is influenced by tumor site, size, histologic features, and surgical margins.

Pleural SFTs have been reported to recur in approximately 9% of cases, whereas extrathoracic tumors exhibit higher rates of local and distant recurrence, estimated at 19%-29% and 13%-34%, respectively [5-7]. Notably, late recurrences occurring more than 10 years after initial resection have been documented, underscoring the importance of prolonged follow-up. These observations highlight the unpredictable natural history of SFTs and the limitations of traditional risk stratification in fully capturing long-term behavior.

## Case presentation

**Figure 1:**
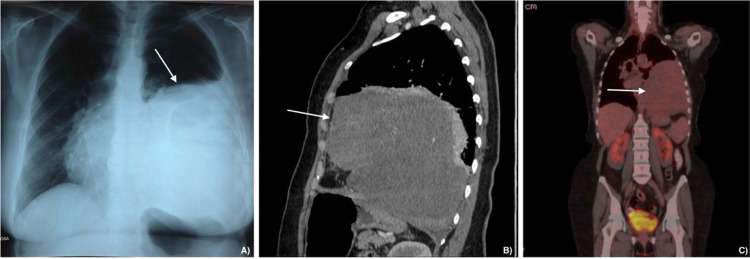
Preoperative imaging of the initial tumor. (A) Chest radiograph (posteroanterior view), (B) computed tomography (CT) sagittal reconstruction, and (C) positron emission tomography-computed tomography (PET-CT) coronal reconstruction of the first tumor, obtained ten years before the current presentation. Imaging demonstrates a large, well-circumscribed, homogeneous opacity occupying the left mid-to-lower hemithorax, with convex lateral margins and effacement of the left cardiac border and hemidiaphragm (silhouette sign), consistent with a pleural-based mass.

An 83-year-old female with a past medical history of tetracycline allergy, appendectomy, hysterectomy, and cholecystectomy presented for routine follow-up. Of note, 12 years earlier, she had developed a persistent cough that prompted a chest radiograph (Figure 1), which revealed a thoracic mass. Further evaluation with computed tomography (CT) confirmed the lesion, and a CT-guided biopsy established the diagnosis of an SFT. She subsequently underwent surgical resection of the mass.

On the current view, a chest CT scan performed on June 5 demonstrated well-distended lungs with diffuse interstitial thickening and small peripheral cystic lesions. In the mediastinum, a lobulated solid-density mass (62 Hounsfield units) was identified in the anterior left compartment, measuring 4.7 × 3.7 cm in axial dimensions, without associated lymphadenopathy. Compared with prior studies, the lesion demonstrated interval growth and increased lobulation, raising concern for neoplasia.

**Figure 2:**
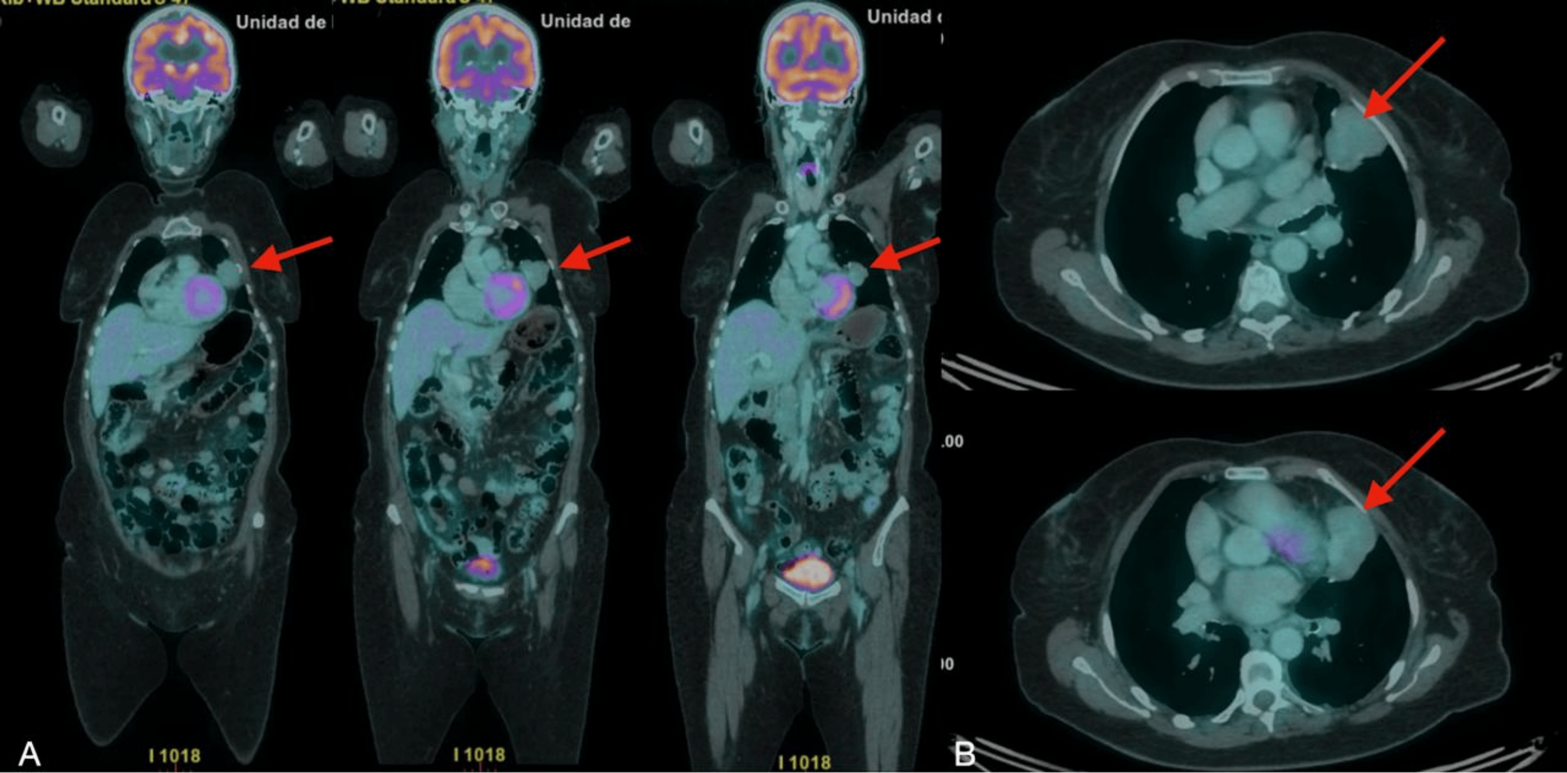
Positron emission tomography-computed tomography (PET-CT) findings of a recurrent solitary fibrous tumor. (A) Coronal fused 18F-fluorodeoxyglucose (18F-FDG) PET-CT images demonstrating a lobulated solid mass involving the lingular and anterior segment of the left upper lobe (red arrows), with low and heterogeneous FDG uptake. (B) Axial CT and fused PET-CT images showing the lesion abutting the pleura and contacting the mediastinum (red arrows), without associated hypermetabolic mediastinal or hilar lymphadenopathy. The maximum standardized uptake value (SUVmax) of the lesion was 2.32.

On the current view, a subsequent positron emission tomography-computed tomography (PET-CT) scan performed on June 30 demonstrated a lobulated solid mass involving the lingular and anterior segment of the left upper lobe, measuring 47 × 36 mm (Figure 2). The lesion abutted the pleura and showed loss of the fat plane with the mediastinum, with peripheral surgical material consistent with prior thoracic intervention. It exhibited low and heterogeneous fluorodeoxyglucose uptake, with a maximum standardized uptake value (SUVmax) of 2.32.

An additional small lobulated pulmonary nodule was identified in the posterior basal segment of the right lower lobe, demonstrating low metabolic activity (SUVmax 1.15). No hypermetabolic mediastinal or hilar lymphadenopathy or distant metabolically active disease was identified. Overall, the findings were considered indeterminate, and correlation with histopathological results was recommended.

Pulmonary function testing confirmed adequate respiratory reserve, and the patient was scheduled for surgical resection. Intraoperatively, the tumor was found to be firmly adherent to the pericardial pleura. Given its location and size, resection was performed through a left thoracoscopy without intraoperative complications. A 24-Fr chest tube and a kardiaspiral drain were placed and subsequently removed on postoperative day 6 after daily radiological follow-up confirmed satisfactory lung expansion. The postoperative course was uneventful, and the patient was discharged home on postoperative day 7.

**Figure 3:**
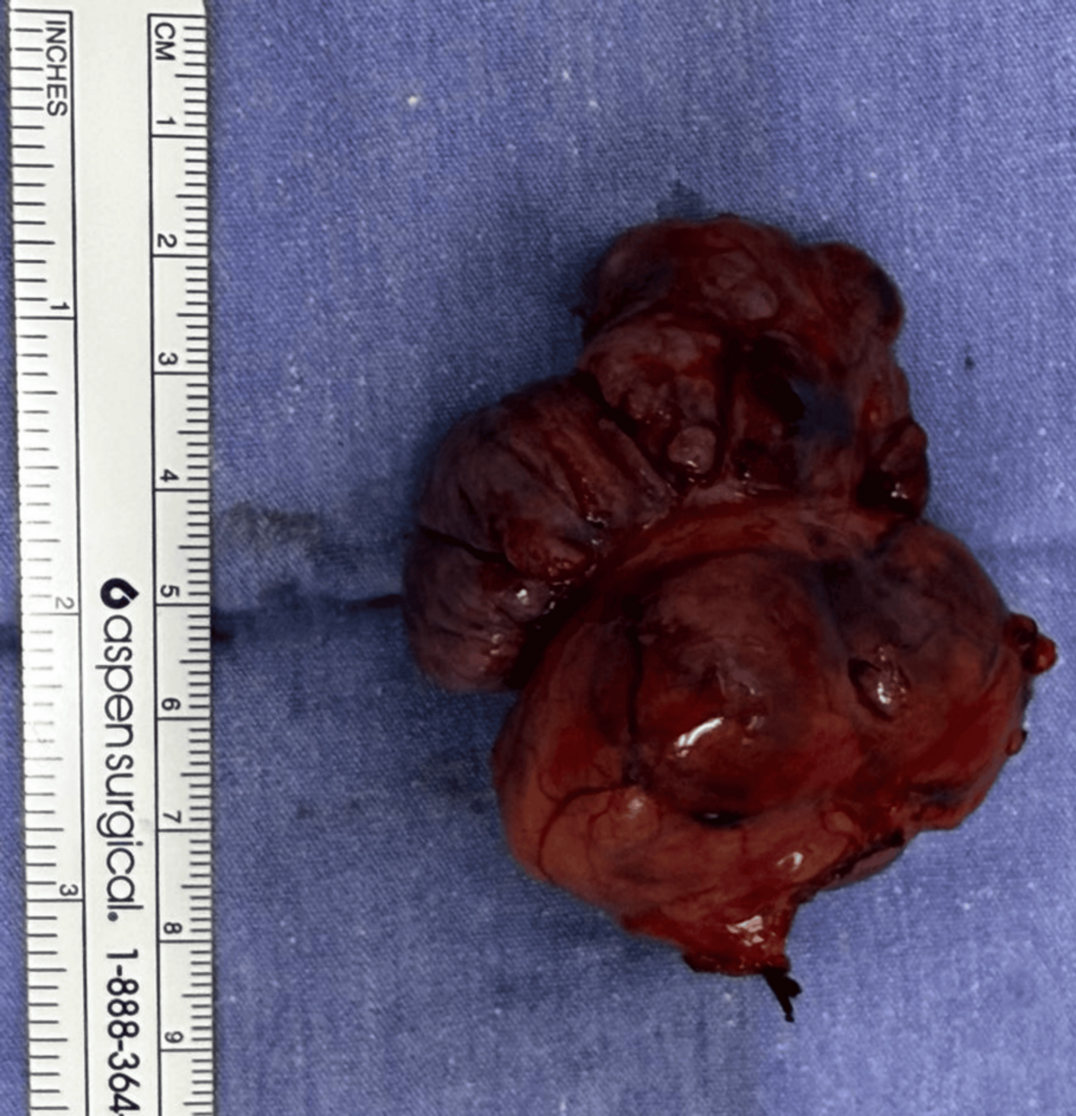
Gross specimen of the resected tumor. The specimen consists of an ovoid, multinodular, well-circumscribed mass measuring 6.5 × 5 cm and weighing 53.4 g. The external surface is lobulated with a reddish-brown appearance. On sectioning, the cut surface is solid, whitish, and homogeneous, findings consistent with a solitary fibrous tumor.

Histopathological analysis of the resected specimen allowed direct comparison with the patient’s prior tumor. The current mass, located in the pericardial pleura, measured 6.5 × 5 cm and weighed 53.4 g (Figure 3). Gross examination revealed a well-circumscribed, ovoid, multinodular lesion with a firm, whitish, homogeneous cut surface. Microscopically, the tumor demonstrated spindle-cell proliferation arranged in a patternless architecture, without evidence of hypercellularity, necrosis, or pleomorphism.

Immunohistochemistry revealed strong nuclear positivity for STAT6 and diffuse CD34 expression, while markers such as S100 protein and muscle-specific actin were negative. The Demicco 2017 risk stratification model was applied, and the tumor scored 2 points, placing it in the low-risk category for recurrence and metastasis.

**Figure 4:**
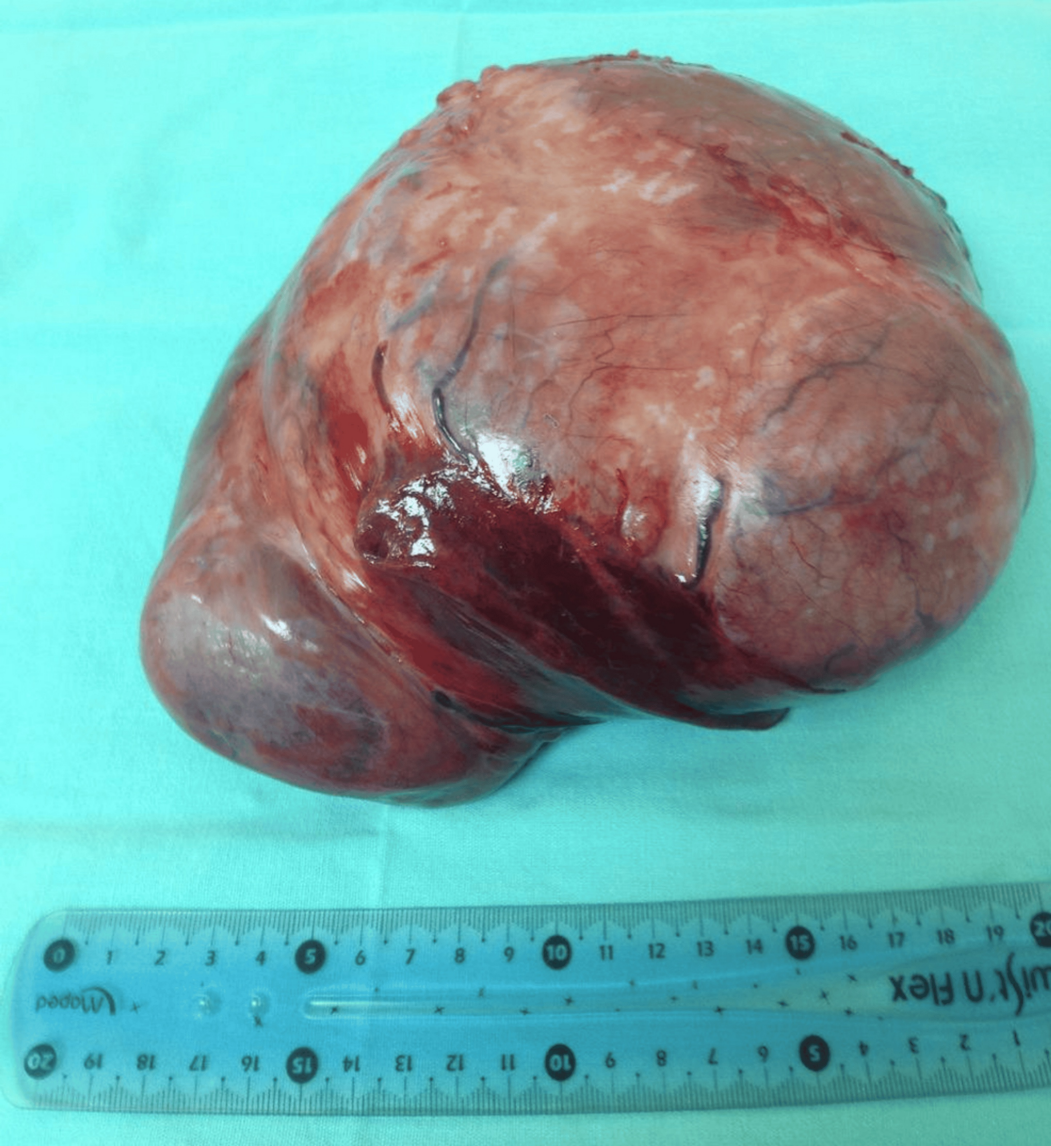
Gross specimen of the pleural solitary fibrous tumor resected 12 years earlier. The tumor measures approximately 20 × 15 cm and appears as a tan-pink, lobulated, well-circumscribed mass with a smooth, glistening surface and focal subcapsular hemorrhage. A ruler is shown for scale.

For comparison, the patient’s previous tumor, resected in 2013, measured 19.3 × 16.2 cm (Figure 4) and showed immunophenotypic positivity for CD34, CD99, and β-catenin, with negativity for cytokeratin AE1/3 and a Ki-67 proliferation index of approximately 5%. These findings confirmed the diagnosis of SFT in both instances, while also illustrating the evolution of diagnostic immunohistochemical approaches over the past decade, particularly the incorporation of STAT6 as a highly specific marker.

## Discussion

SFTs are rare mesenchymal neoplasms that can arise across a wide age spectrum, with peak incidence reported in the fifth and sixth decades of life [8,9]. Although SFTs may originate in virtually any anatomic location, the pleura represents the most common site, accounting for approximately 30% of cases. Other frequently involved locations include the meninges, abdominal cavity, trunk, extremities, and head and neck region [10]. Despite their often indolent presentation, SFTs are characterized by an unpredictable clinical course, with the potential for local recurrence and distant metastasis even after complete resection.

Historically, SFTs were initially described as pleural tumors of presumed mesothelial origin by Klemperer and Rabin in 1931 [11]. Subsequent advances in histopathology, immunohistochemistry, and molecular biology have firmly established their mesenchymal origin, distinguishing them from mesotheliomas and other pleural neoplasms [12,13]. Clinically, SFTs may remain asymptomatic for prolonged periods or present with compressive symptoms depending on size and location. Thoracic SFTs most commonly manifest with cough, dyspnea, chest pain, or hemoptysis, whereas paraneoplastic syndromes, such as hypertrophic osteoarthropathy, are less frequently observed [1,14].

Radiologic evaluation plays a crucial role in the detection and surgical planning of SFTs; however, there are no radiologic pathognomonic features. On computed tomography, these tumors typically appear as well-circumscribed, isodense soft-tissue masses, often demonstrating heterogeneous contrast enhancement related to their vascularity [15]. Magnetic resonance imaging commonly reveals areas of low signal intensity on both T1- and T2-weighted sequences, reflecting dense collagenous stroma. PET-CT may offer complementary information, particularly in assessing aggressive behavior or metastatic disease, although low metabolic activity is frequently observed in indolent tumors, as demonstrated in the present case [8]. Consequently, low FDG uptake should not exclude the diagnosis of SFT or malignant potential.

This case underscores the unpredictable biological behavior of SFTs, including the potential for clinically significant recurrence more than a decade after apparently curative resection, even in the setting of low FDG uptake.

Grossly, SFTs are typically well-circumscribed and partially encapsulated, with a firm, whitish, multinodular cut surface. Microscopically, they are composed of spindle to oval cells arranged in a so-called *patternless pattern*, embedded within a variably collagenous stroma and intersected by thin-walled, branching (*staghorn*) vessels [10,15,16]. Immunohistochemistry remains the cornerstone of diagnosis. While classic markers such as CD34, Bcl-2, and CD99 are frequently expressed, nuclear positivity for signal transducer and activator of transcription 6 (STAT6), resulting from NAB2-STAT6 gene fusion, has emerged as the most sensitive and specific diagnostic marker for SFTs [2,8,17].

Given the heterogeneous behavior of SFTs, several prognostic models have been developed to estimate the risk of recurrence and metastasis. The Demicco risk stratification model and its modified version (mDemicco) are among the most widely validated tools, incorporating patient age, tumor size, mitotic activity per 10 high-power fields, and necrosis to classify tumors into low-, intermediate-, and high-risk categories (Table 1), with corresponding predicted metastatic risks (Table 2) [18-20]. These models provide valuable guidance but do not fully capture the long-term biological behavior of SFTs.

**Table 1:** Demicco and modified Demicco (mDemicco) risk stratification models for solitary fibrous tumors. The modified Demicco (mDemicco) model incorporates tumor necrosis as an additional variable to improve prognostic accuracy. HPF, high-power fields

Variable	Criteria	Score
Patient age (years)	<55	0
	≥55	1
Tumor size (cm)	<5	0
	5 to <10	1
	10 to <15	2
	≥15	3
Mitotic activity (per 10 HPF)	0	0
	1 to 3	1
	≥4	2
Necrosis (%)	0%	0
	>0%	1

**Table 2:** Risk group classification and predicted metastatic risk according to the Demicco and modified Demicco models. Risk categories are based on cumulative scoring of age, tumor size, mitotic activity, and necrosis, as described by Demicco et al. and subsequent validation studies [18-20].

Total score	Risk group	Predicted metastatic risk
0-3 points	Low	Minimal to none at 10 years
4-5 points	Intermediate	~10% to 20% metastasis at 10 years
6-7 points	High	Up to 73% metastasis within five years

In the present case, the initial tumor resected in 2013 was large and retrospectively classified as intermediate risk, whereas the second lesion resected twelve years later demonstrated low-risk features according to the Demicco model. The long disease-free interval raises an important diagnostic dilemma: late recurrence versus a metachronous SFT. Late recurrences have been documented more than a decade after complete resection and may reflect dormant microscopic disease with prolonged latency [18,19]. Alternatively, the development of a new tumor in an anatomically distinct but adjacent thoracic location, sharing similar histologic and immunophenotypic features, supports the possibility of a metachronous neoplasm [20]. Distinguishing between these entities is inherently challenging, as nearly all SFTs harbor NAB2-STAT6 gene fusions, and only detailed molecular clonality analyses could provide definitive differentiation - studies that are rarely performed in routine clinical practice.

Regardless of classification, this case highlights the limitations of existing prognostic models and underscores the unpredictable natural history of SFTs. Complete surgical resection with negative margins remains the cornerstone of treatment and offers the best chance for durable disease control [8,12,13,16]. Adjuvant therapy is generally reserved for high-risk or incompletely resected tumors. In advanced or unresectable disease, antiangiogenic agents, including tyrosine kinase inhibitors, have demonstrated superior activity compared with conventional cytotoxic chemotherapy, which is typically associated with limited benefit [8,16].

Overall, this case emphasizes that SFTs, even those classified as low risk, may exhibit late and unexpected disease manifestations. Long-term, and potentially lifelong, surveillance should therefore be considered an integral component of management for patients with thoracic SFTs, irrespective of initial risk stratification.

## Conclusions

This case illustrates the unpredictable biological behavior of SFTs, demonstrating that clinically significant disease may emerge more than a decade after apparently curative surgical resection. The prolonged disease-free interval in our patient raises the diagnostic challenge of distinguishing late recurrence from a metachronous tumor, a distinction that remains difficult in routine practice due to shared histologic and molecular features among SFTs. Importantly, this case highlights the limitations of current risk stratification models and metabolic imaging in fully predicting long-term behavior, particularly in tumors classified as low risk. Complete surgical resection with negative margins remains the cornerstone of management; however, long-term, and potentially lifelong, surveillance should be considered essential for patients with thoracic SFTs to ensure early detection of recurrence and optimize clinical outcomes.
